# Routine Surveillance and Vaccination on a University Campus During the Spread of the SARS-CoV-2 Omicron Variant

**DOI:** 10.1001/jamanetworkopen.2022.12906

**Published:** 2022-05-18

**Authors:** Genevive R. Meredith, Diego G. Diel, Peter I. Frazier, Shane G. Henderson, Gary A. Koretzky, Jiayue Wan, Lorin D. Warnick

**Affiliations:** 1 Department of Public and Ecosystem Health, College of Veterinary Medicine, Cornell University, Ithaca, New York; 2 Department of Population Medicine and Diagnostic Sciences, College of Veterinary Medicine, Cornell University, Ithaca, New York; 3 Department of Operations Research and Information Engineering, College of Engineering, Cornell University, Ithaca, New York; 4 Provost’s Office, Cornell University, Ithaca, New York

## Abstract

This case series study of COVID-19 data from a US university examines the effectiveness of various vaccination, testing, and surveillance measures to mitigate spread of the Omicron variant.

## Introduction

As SARS-CoV-2 was detected in the US, emergency public health measures took effect, including shutting down schools.^1^ As prevention and control measures improved, emergency response policies were rolled back.^1^ Cornell University opened for residential instruction in Fall 2021 using an extensive testing, contact tracing, and isolation program in partnership with the Tompkins County Health Department (Table).^2^ Vaccination was mandated for all students and encouraged for employees. Masks were required on-campus, and isolation orders and contact tracing occurred within hours of any positive result. We hypothesized that these measures would limit COVID-19 spread on campus and sought to monitor this with a case-series study of university testing records.

**Table.** Public Health Measures Implemented in Fall 2021 to Mitigate COVID-19 Transmission and Morbidity on Campus

Public health measures	Focus	Outcomes
Mask wearing Required inside all buildings on campus, all semester (except in private office space or in designated eating areas with distancing)	Prevention	Layer of protection against COVID-19 transmission
Vaccination Required for students Strongly encouraged for employees	Prevention, mitigation	Protection against COVID-19 transmission and/or impact: 97.9% of campus fully vaccinated
Daily symptom screening and telehealth appointments (for questions or concerns with symptoms) Required for employees Strongly encouraged for students	Detection, mitigation	Layer of protection against COVID-19 transmission and/or impact
Free mandatory PCR surveillance 100% of undergraduates 28.5% of graduate and professional students 20.9% of employees	Detection	Early detection of COVID-19; detection of asymptomatic/mildly symptomatic cases: August 18-December 31—mean tests/d, 3335; median, 3109 tests/d (range, 14-6959 tests/d) >60% of campus community tested each wk Testing noncompliance monitored; nudges issued; noncompliance resulted in limits to campus resources
Free PCR testing to anyone, 6 d per wk Multiple locations on campus, in community		
Expedient testing and follow-up Test results within 24-48 h Case investigation within hours of test resulting Contact tracing within hours of test resulting Contact notification within hours of case investigation	Mitigation, prevention	Within 24-48 h of sample: Test result in portal Phone-based case support to assure understanding of positive result, connection to health care resources, isolation instructions, isolation support (off-campus hotel if needed, food if needed, academic or work leave plans), and to initiate contact tracing Contacts notified; instructions provided to monitor symptoms, access testing, quarantine (if symptomatic and/or not fully vaccinated)
Integrated data system (with county health department, student health, local hospitals) Testing registration Push message reminders Test resulting Case management	Detection, mitigation	Case data inclusive of positive samples taken/tested off-campus: Support for isolation, workplace leave, and academic accommodations provided to individuals testing positive Contact tracing Contact notification

Abbreviation: PCR, polymerase chain reaction.

## Methods

For the Fall semester (August 26 through December 18, 2021), all undergraduates (15 503 students), 2873 graduate students (28.5%), and 2803 employees (20.9%) were required to register for and participate at least once a week in free, on-campus polymerase chain reaction COVID-19 surveillance testing.^2^ Using a case series approach, all deidentified university surveillance data (ie, test registration, result) were reviewed daily to detect sentinel events and outbreaks and to guide public health responses; testing compliance rate, test positivity rate, and incidence were monitored. Routinely, positive specimens were sequenced for genetic characteristics. As part of Cornell University’s institutional operations, this public health surveillance effort was not subject to institutional review board review, and informed consent was not needed because data were nonidentifiable counts. This study followed the reporting guideline for case series.

## Results

When students returned to campus (mid-August 2021), reentry testing was used to identify COVID-19 cases (Figure).^3^ Isolation, case investigation, contact tracing, quarantine, and targeted supplemental testing limited the outbreak to 480 cases (August 23 to September 10: mean [SD] 22.9 [18.8] cases/d). Thereafter, routine surveillance and public health measures limited transmission (September 12 to November 27: students, 1.9 [2.2] cases/d; employees, 2.4 [2.5] cases/d; 330 total cases; 0.1% positivity) (Table).

**Figure.**
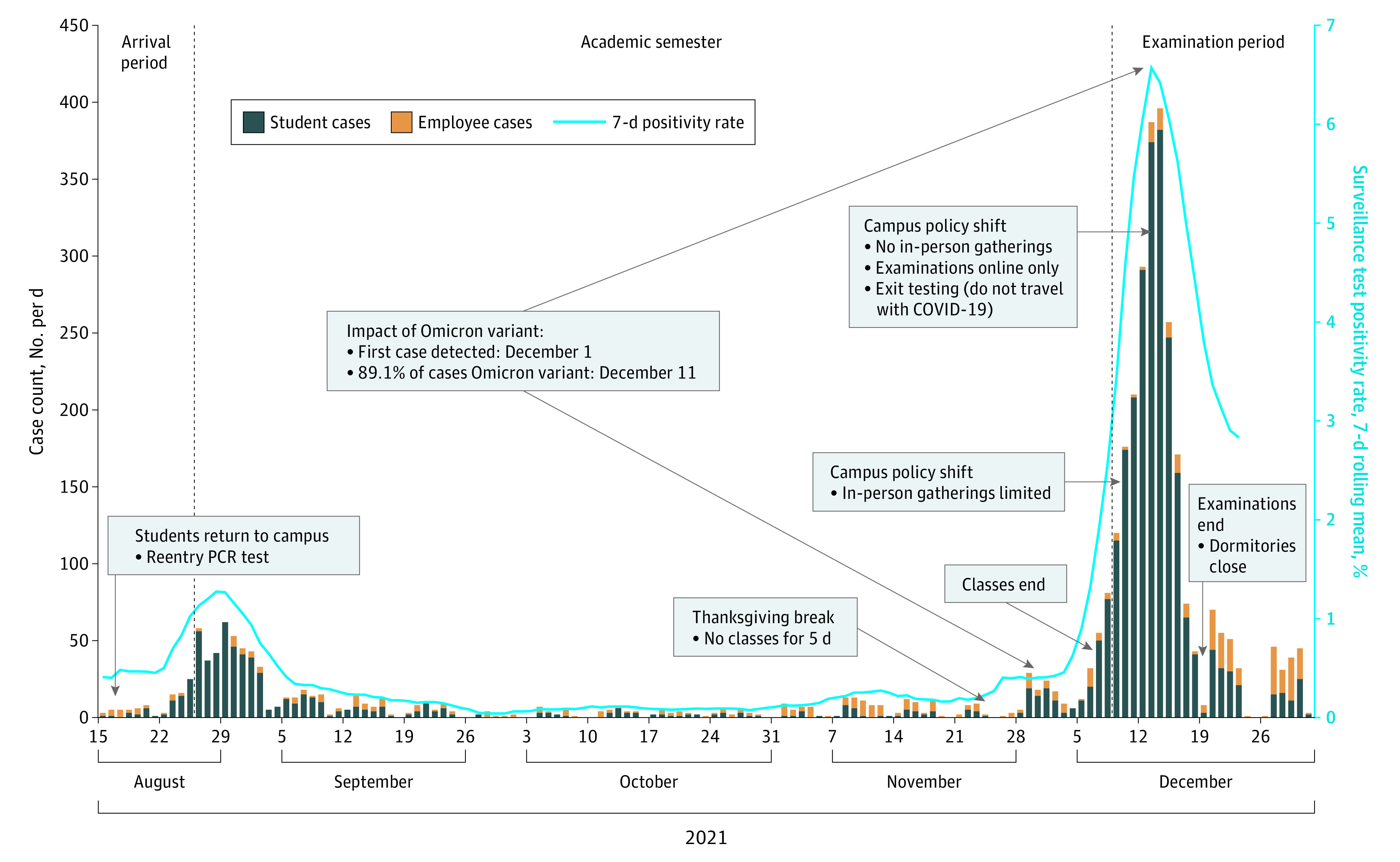
Identified COVID-19 Case Trends and Key Events, Cornell University, Fall 2021 PCR indicates polymerase chain reaction.

After Cornell’s 5-day Thanksgiving break, surveillance outcomes changed dramatically among students (Figure): 75 cases from November 28 to December 4 (mean [SD], 10.7 [6.9] cases/d; 0.5% positivity), 655 from December 5 to December 11 (93.6 [75.7] cases/d; 2.9% positivity), and 1559 from December 12 to December 18 (222.7 [138.7] cases/d; 5.7% positivity). Support teams helped cases isolate safely, investigation identified exposures, and contact tracing identified contacts who were instructed to monitor for symptoms, test, and/or quarantine.

From November 28 to December 31, 2797 COVID-19 cases were identified (mean [SD], 82.3 [82.4] cases/d; 3.1% positivity; 89.0% students, 11.0% employees), eclipsing previously measured incidence. Most cases (82.2%) reported mild symptoms (no reported hospitalizations). Despite high vaccination rates (97.9% of campus^3^), 98.6% of cases were breakthrough infections, and proportionately more named close contacts who became COVID-positive in this period (22.6%) than previously (4.4% between August 23 and November 27). Something had clearly changed in the university setting, as similar outbreaks were not yet being seen in the off-campus community or neighboring counties.^4^

From mid-November, positive samples were screened for *S* gene target failure as a marker of variant Omicron.^5^ Whole genome sequencing confirmed the presence of Omicron in samples from December 1 (1 sample), December 2 (1 sample), December 3 (2 samples), and December 4 (4 samples). By December 11, 155 of the 174 positive samples (89.1%) were confirmed as Omicron; the Delta variant was detected in the remaining samples.

Given identification of Omicron and the noted speed of transmission, on December 10 university leadership limited in-person interactions, and on December 14 student gatherings were prohibited, examinations were moved online, and an exit testing process was implemented.^2^ The de-densification process decreased student cases numbers,^3^ but incidence among people who stayed locally remained higher than before Thanksgiving (December 26 to December 31: students, 11.5 [9.4] cases/d; employees, 16.0 [12.9] cases/d; 4.8% positivity).

## Discussion

The Omicron variant is highly transmissible, particularly in high-density social settings.^5,6^ Based on analysis of routinely collected population surveillance data, Cornell’s experience shows that traditional public health interventions were not a match for Omicron. While vaccination protected against severe illness, it was not sufficient to prevent rapid spread, even when combined with other public health measures including widespread surveillance testing. Generalizability of the study finding might be limited due to the demographics of its sample (the majority of participants were undergraduate students) and by the study’s single institutional setting. As SARS-CoV-2 continues to adapt, surveillance and case-series studies that look across different populations and settings will be helpful in identifying sentinel events and guiding actions to mitigate harm.
